# Characterization of the gut microbiome of patients with *Clostridioides difficile* infection, patients with non–*C. difficile* diarrhea, and *C. difficile*–colonized patients

**DOI:** 10.3389/fcimb.2023.1130701

**Published:** 2023-04-12

**Authors:** Silvia Vázquez-Cuesta, Laura Villar, Nuria Lozano García, Ana I. Fernández, María Olmedo, Luis Alcalá, Mercedes Marín, Patricia Muñoz, Emilio Bouza, Elena Reigadas

**Affiliations:** ^1^ Department of Clinical Microbiology and Infectious Diseases, Hospital General Universitario Gregorio Marañón, Madrid, Spain; ^2^ Instituto de Investigación Sanitaria Gregorio Marañón, Madrid, Spain; ^3^ Biochemistry and Molecular Biology Department, Faculty of Biology, Universidad Complutense de Madrid (UCM), Madrid, Spain; ^4^ Centro de Investigación Biomédica en Red (CIBER) de Enfermedades Respiratorias (CIBERES CB06/06/0058), Madrid, Spain; ^5^ Medicine Department, School of Medicine, Universidad Complutense de Madrid (UCM), Madrid, Spain; ^6^ European Society of Clinical Microbiology and Infectious Diseases (ESCMID) Study Group for Clostridioides difficile (ESGCD), Basel, Switzerland

**Keywords:** CDI, *C. difficile*, microbiome, R-CDI, 16S rRNA

## Abstract

**Introduction:**

*Clostridioides difficile* infection (CDI) is the main cause of nosocomial diarrhea in developed countries. A key challenge in CDI is the lack of objective methods to ensure more accurate diagnosis, especially when differentiating between true infection and colonization/diarrhea of other causes. The main objective of this study was to explore the role of the microbiome as a predictive biomarker of CDI.

**Methods:**

Between 2018 and 2021, we prospectively included patients with CDI, recurrent CDI (R-CDI), non-CDI diarrhea (NO-CDI), colonization by *C. difficile*, and healthy individuals. Clinical data and fecal samples were collected. The microbiome was analyzed by sequencing the hypervariable V4 region of the 16S rRNA gene on an Illumina Miseq platform. The mothur bioinformatic pipeline was followed for pre-processing of raw data, and mothur and R were used for data analysis.

**Results:**

During the study period, 753 samples from 657 patients were analyzed. Of these, 247 were from patients with CDI, 43 were from patients colonized with *C. difficile*, 63 were from healthy individuals, 324 were from NOCDI, and 76 were from R-CDI. We found significant differences across the groups in alpha and beta diversity and in taxonomic abundance. We identified various genera as the most significant biomarkers for CDI (*Bacteroides, Proteus, Paraprevotella, Robinsoniella*), R-CDI (*Veillonella, Fusobacterium, Lactobacillus, Clostridium sensu stricto I*), and colonization by *C. difficile* (*Parabacteroides, Faecalicoccus, Flavonifractor, Clostridium XVIII*).

**Discussion:**

We observed differences in microbiome patterns between healthy individuals, colonized patients, CDI, R-CDI, and NOCDI diarrhea. We identified possible microbiome biomarkers that could prove useful in the diagnosis of true CDI infections. Further studies are warranted.

## 1 Introduction

*Clostridioides difficile* infection (CDI) is the main cause of nosocomial diarrhea in developed countries (Dubberke and Olsen, 2012). The clinical severity of CDI can be classified as follows: absence of symptoms; mild, moderate, and severe disease; pseudomembranous colitis; and toxic megacolon, sepsis, and death (Rupnik et al., 2009).

Risk factors associated with CDI include advanced age, hospital stay, treatment with proton pump inhibitors, and prolonged antibiotic treatment or treatment with multiple antibiotics (Bignardi, 1998). The risk factor most closely associated with disease is use of antibiotics, as it is directly related to dysbiosis of the gut microbiota, which enables germination of and colonization by *C. difficile* (Theriot and Young, 2015; Theriot et al., 2016; Thanissery et al., 2017). Dysbiosis caused by antibiotic treatment is reflected in decreased diversity and loss of specific taxa, both of which alter the variety and quantity of metabolites present in the gut (Jernberg et al., 2007; Dethlefsen et al., 2008; Jernberg et al., 2010; Britton and Young, 2014).

Age also plays an important role in the risk of CDI, as the gut microbiota becomes less diverse with age. In addition, the presence of chronic diseases and the use of multiple drugs, including antibiotics, considerably affects the microbiota, thus increasing the risk of colonization by *C. difficile* in elderly patients (Odamaki et al., 2016; Gao et al., 2018).

Because of the close relationship between gut microbiota and *C. difficile*, guidelines recommend fecal microbiota transplantation for recurrent episodes of CDI in order to restore patients’ gut microbiota and prevent further recurrences (McDonald et al., 2018). However, more preventive strategies are needed.

The considerable associated health and economic burden of CDI calls for novel strategies by which CDI can be prevented in susceptible patients (Heimann et al., 2018; Reigadas Ramirez and Bouza, 2018). Profiling differences between the gut microbiota of patients with CDI, that of healthy individuals, and that of individuals with diarrhea due to other causes could help us to predict which patients are at immediate risk for CDI, which will progress better or worse, and which will experience recurrence of CDI.

One of the challenges in diagnosing CDI is that of being able to distinguish between patients with true CDI and those who are colonized (Planche and Wilcox, 2015). The search for new methods for the detection of *C. difficile* and for distinguishing true infection from colonization in diarrhea of other causes is ongoing (Kelly et al., 2020; Sandlund et al., 2020; Ng et al., 2021). Profiling the microbiome and identifying possible microbiome biomarkers could help us differentiate between infection and colonization by *C. difficile* and diarrhea of other causes (NOCDI) (Planche and Wilcox, 2015).

The main objective of this study was to explore the role of the microbiome as a predictive biomarker of true CDI.

## 2 Methods

### 2.1 Setting, design, and study population

The study was carried out at Hospital General Universitario Gregorio Marañón in Madrid (Spain), a 1,350-bed tertiary university hospital. Determination of toxigenic *C. difficile* is routinely performed on all loose stool samples from patients older than 2 years. The microbiology laboratory receives samples both from the hospital itself and from 13 outpatient centers in the same area.

We conducted a prospective study from 2018 to 2021. We enrolled patients whose stool samples were sent to the microbiology laboratory and healthy individuals. We collected clinical data and diagnostic fecal samples from all patients. The participants were classified into the following groups: healthy individuals, patients with primary CDI (CDI), patients with recurrent CDI (R-CDI), patients colonized by toxigenic *C. difficile* (colonized) and patients with diarrhea who tested negative for *C. difficile* (NOCDI).

### 2.2 Definitions

An episode of CDI was defined as the presence of a positive toxigenic CDI test, together with diarrhea (≥3 loose stools in 24 hours) or findings of pseudomembranous colitis by colonoscopy, following the definitions set out in the guidelines of the Society for Healthcare Epidemiology of America (SHEA) and Infectious Diseases Society of America (IDSA) (McDonald et al., 2018).

We considered a patient to be colonized with toxigenic *C. difficile* when he/she tested positive for toxigenic *C. difficile* but did not meet clinical criteria for CDI, as defined above.

The severity of the CDI episode was defined according to the SHEA and IDSA guidelines (McDonald et al., 2018).

R-CDI was defined as CDI recurring within 8 weeks of a previous episode, provided the symptoms of the previous episode resolved after completion of initial treatment (van Prehn et al., 2021). Having new symptoms and a positive sample after 60 days was considered a new episode.

Death was considered CDI-related when there were no other attributable causes and/or it occurred within 10 days after the diagnosis of CDI and/or was due to known complications of CDI.

Healthy individuals were defined as those who did not meet any of the following criteria: body mass index lower than 17 or higher than 30, any type of disease including microbiota-related disease (cholelithiasis, colorectal cancer, hepatic encephalopathy, idiopathic constipation, inflammatory bowel disease, irritable bowel syndrome, familial Mediterranean fever, gastric lymphoma or carcinoma, arthritis, asthma, atopy, dermatitis, psoriasis, autoimmune disease, fatigue syndrome, diabetes mellitus, hypercholesterolemia, idiopathic thrombocytopenic purpura, myocardial ischemia, metabolic syndrome, behavioral disorders, multiple sclerosis, myoclonus dystonia, non-alcoholic fatty liver disease, oxalate kidney stones, Parkinson’s disease), gastrointestinal disorders, immunologic disease, immunocompromise, alcohol intake higher than 50 g/day, and use of antibiotics, probiotics, immunosuppressants, proton pump inhibitors, or vaccines in the previous three months.

As for microbiome-related definitions, we considered richness as the number of different species found in a sample (Whittaker et al., 2001). Evenness was defined as the degree to which different species are similar or uniform in abundance. Diversity indicated the degree of species richness and abundance, where alpha diversity referred to the diversity within an individual and beta diversity referred to the difference in diversity between individuals (Whittaker et al., 2001).

### 2.3 Detection of *C. difficile*

Samples were processed using a rapid detection kit for toxigenic *C. difficile*. This rapid test involves detection of the antigen by immunochromatography (C Diff Quik-Chek Complete assay, TechLab, Blacksburg, VA, USA) and a real-time polymerase chain reaction (PCR) of the toxin B gene (XpertTM*C. difficile* Assay, GeneXpert, Cepheid, Sunnyvale, CA, USA).

In addition, all samples were cultured on *C. difficile* selective agar (bioMeriéux, Marcy l’Etoile, France). Suspected toxigenic *C. difficile* colonies were confirmed using immunochromatography (C Diff Quik-Chek Complete assay, TechLab, Blacksburg, VA, USA).

### 2.4 Clinical data

The demographic data collected included age and sex. Regarding clinical data, underlying conditions were recorded using the McCabe and Jackson score for prognosis of underlying diseases (McCabe and Jackson, 1962); comorbidity was graded according to the Charlson comorbidity index (Charlson et al., 1987). Other clinical data collected included antibiotic treatment, proton pump inhibitor treatment, nasogastric tube use, mechanical ventilation, surgery, and chemotherapy or radiotherapy in the month prior to CDI diagnosis. For the CDI episode, data on severity, treatment received, treatment failure, recurrence, mortality, and CDI-related mortality were recorded.

### 2.5 Sample processing

Immediately upon receipt, the fecal samples were homogenized, aliquoted, and stored at −80°C until the day of analysis. Total DNA was extracted from fecal samples using the Qiagen Fast QiaAmp DNA Stool Mini Kit (QIAGEN, Valencia, CA, USA) according to the manufacturer’s protocol with the inclusion of a physical lysis step. The sample was lysed in FastPrep-24 (MPBio, Derby, UK) with lysis matrix E tubes (MPBio, Derby, UK) twice at 6.5 m/s for 45 seconds. The hypervariable V4 region of the 16s rRNA gene was amplified by polymerase chain reaction with 515-806 primers tailed with sequences to incorporate Illumina flow cell adapters and indexing barcodes (Illumina, San Diego, USA).

Primer dimers and low-molecular-weight products were removed using Agencourt Ampure Beads (Beckman Coulter, Spain) and samples were quantified and quality checked for amplicon size using the 4200 TapeStation (Agilent Technologies, Santa Clara, CA, USA). Amplicons were pooled in equimolar amounts and sequenced (2 × 250) on an Illumina Miseq system (Illumina, San Diego, USA) according to standard protocols.

### 2.6 Data analysis

The raw data were pre-processed and grouped by operational taxonomic units (OTUs) with 97% similarity and classified taxonomically using mothur software (Patrick D. Schloss, PhD, ^©^ 2019, Michigan, USA) and SILVA and RDP Ribosomal Database Project databases. Species richness (OTUs observed), evenness (Pielou index), alpha diversity (Shannon index, inverse Simpson index), and beta diversity (Bray-Curtis distance, unweighted unifrac distance) were analyzed using mothur and R software (R Project for Statistical Computing) (R Core Team, 2021, Vienna, Austria).

Statistical analyses were performed with R (R Core Team, 2021, Vienna, Austria). Frequencies were calculated for qualitative variables, and proportions were calculated with their 95% confidence interval following a binomial distribution. For quantitative variables, the median and interquartile range (IQR) or mean and standard deviation (SD) were calculated. Microbiota analyses were performed with R using the Bioconductor packages phyloseq, microbiome, microbiomeStat, vegan, DESeq2, and microeco.

Biomarkers were found using linear discriminant analysis effect size (LEfSe) and random forest (RF). For the LEfSe analysis, the Kruskal-Wallis non-parametric factorial rank sum test was first performed, then the pairwise groups were analyzed using the unpaired Wilcoxon rank sum test. Following these steps, linear discrimination analysis (LDA) was used. LDA score higher than 3 was used. For random forest analysis bootstrapt test number selected was 30 and 1000 trees to grow. Method for adjusted p-values was false discovery rate and Mean Decrease Gini was selected as the indicator value in the analysis.

Differences between groups were determined using the χ2 test; differences for continuous variables were assessed using the *t* test. The Mann-Whitney test was used for non-normal distributions. The normality of the distribution of continuous variables was tested using the Kolmogorov-Smirnov test with the Lilliefors correction.

## 3 Results

During the study period, a total of 753 samples from 657 patients were analyzed. Of these, 247 samples were obtained from 233 patients with CDI, 43 samples from 40 patients colonized with *C. difficile*, 63 samples from 63 healthy individuals, 324 samples from 264 patients with diarrhoea without *C. difficile*; and 76 samples from 57 R-CDI.

### 3.1 Demographic and clinical characteristics

The median age of the patients was around 70 years, with more females in all groups except NOCDI, as follows: CDI, 56.2% (131/233); colonized, 52.5% (21/40); NOCDI, 49.2% (130/264); and R-CDI, 68.4% (39/57) (p=0.053). Most healthy individuals were female (52.4%), and the median age was 32 years (range, 0-76). The most common underlying conditions were cardiovascular, metabolic, endocrine, nephro-urological, and gastrointestinal diseases (**Table 1**). The median Charlson comorbidity index was 4 (IQR: 2-6) in the CDI, R-CDI, and NOCDI groups and 4 (IQR: 2.8-5.2) in the colonized group. The percentage of patients with underlying diseases related to microbiota abnormalities was high in all groups, although it was higher in R-CDI: colonized, 60.0% (24/40); NOCDI, 63.6% (168/264); CDI, 67.4% (157/233); and R-CDI, 80.7% (46/57). The most common underlying diseases were diabetes mellitus and myocardial ischemia (**Table 1**). The lowest number of immunosuppressed patients was found among colonized patients: 32.5% (13/40) vs CDI with 36.9% (86/233), NOCDI with 49.6% (131/264), and R-CDI with 43.9% (25/57) (p=0.018) (**Table 1**).

**Table 1:** Clinical characteristics of patients excluded healthy subjects.

	CDI N 229	COLONIZED N 42	NOCDI N 264	RCDI N 53	p.value
**INSTITUTIONALISED PATIENT**	17 (7.4%)	4 (9.5%)	17 (6.5%)	4 (7.5%)	0.901
N-Miss	0	0	1	0	
**HIV**	10 (4.4%)	1 (2.4%)	8 (3.0%)	1 (1.9%)	0.738
**SOLID ORGAN TRANSPLANT**	30 (13.1%)	6 (14.3%)	54 (20.5%)	6 (11.3%)	0.105
**MALIGNANCY**	50 (21.8%)	6 (14.3%)	80 (30.3%)	14 (26.4%)	0.054
**CARDIOLOGICAL DISEASE**	171 (74.7%)	29 (69.0%)	173 (65.5%)	45 (84.9%)	0.015
**PULMONARY DISEASE**	56 (24.5%)	12 (28.6%)	48 (18.2%)	13 (24.5%)	0.23
**GASTROINTESTINAL DISEASE**	79 (34.5%)	16 (38.1%)	101 (38.3%)	26 (49.1%)	0.267
**LIVER DISEASE**	52 (22.7%)	7 (16.7%)	62 (23.5%)	15 (28.3%)	0.609
**HEMATOLOGIC MALIGNANCIES**	40 (17.5%)	10 (23.8%)	58 (22.0%)	12 (22.6%)	0.556
**ENDOCRINE DISEASE**	98 (42.8%)	20 (47.6%)	108 (40.9%)	24 (45.3%)	0.825
**METABOLIC DISEASE**	111 (48.5%)	18 (42.9%)	105 (39.8%)	28 (52.8%)	0.146
**INFECTIOUS DISEASE**	29 (12.7%)	3 (7.1%)	26 (9.8%)	3 (5.7%)	0.375
**ALERGIC DISEASE**	4 (1.7%)	1 (2.4%)	5 (1.9%)	1 (1.9%)	0.994
**RHEUMATIC DISEASE**	58 (25.3%)	11 (26.2%)	60 (22.7%)	16 (30.2%)	0.68
**NEUROLOGICAL DISEASE**	68 (29.7%)	15 (35.7%)	67 (25.4%)	17 (32.1%)	0.418
**NEPHROUROLOGICAL DISEASE**	89 (38.9%)	23 (54.8%)	91 (34.5%)	26 (49.1%)	0.03
**IMMUNE-MEDIATED DISEASE**	10 (4.4%)	3 (7.1%)	18 (6.8%)	5 (9.4%)	0.469
**NUMBER OF DISEASE** Median (Q1, Q3)	5.0 (3.0, 6.0)	4.5 (3.0, 6.0)	4.0 (3.0, 6.0)	5.0 (4.0, 7.0)	0.008
**CHARLSON COMORBIDITY INDEX** Median (Q1, Q3)	4.0 (2.0, 6.0)	3.0 (2.0, 5.0)	4.0 (2.0, 6.0)	4.0 (2.0, 6.0)	0.479
**MICROBIOTA DYSBIOSIS RELATED DISEASE**	153 (66.8%)	25 (59.5%)	168 (63.6%)	44 (83.0%)	0.039
DIABETES MELLITUS	73 (47.7%)	13 (52.0%)	82 (48.8%)	16 (36.4%)	0.476
COLELITHIASIS	21 (13.7%)	0 (0.0%)	17 (10.1%)	9 (20.5%)	0.061
MYOCARDIAL ISCHEMIA	37 (24.2%)	11 (44.0%)	34 (20.2%)	12 (27.3%)	0.071
AUTOIMMUNE DISEASE	5 (3.3%)	3 (12.0%)	12 (7.1%)	1 (2.3%)	0.148
ASTMA	15 (9.8%)	3 (12.0%)	10 (6.0%)	6 (13.6%)	0.319
ATOPIA	0 (0.0%)	1 (4.0%)	1 (0.6%)	0 (0.0%)	0.072
PSORIASIS	3 (2.0%)	0 (0.0%)	2 (1.2%)	0 (0.0%)	0.691
GASTRIC LYMPHOMA OR CARCINOMA	1 (0.7%)	0 (0.0%)	3 (1.8%)	0 (0.0%)	0.598
COLORRECTAL CARCINOMA	12 (7.8%)	0 (0.0%)	16 (9.5%)	5 (11.4%)	0.375
IRRITABLE BOWEL	5 (3.3%)	1 (4.0%)	7 (4.2%)	3 (6.8%)	0.778
INFLAMMATORY BOWEL DISEASE	14 (9.2%)	1 (4.0%)	20 (11.9%)	0 (0.0%)	0.076
IDIOPATHIC THROMBOCYTOPENIC PURPURA	2 (1.3%)	0 (0.0%)	1 (0.6%)	1 (2.3%)	0.715
SKIN DISEASE	2 (1.3%)	0 (0.0%)	1 (0.6%)	1 (2.3%)	0.715
PARKINSON DISEASE	10 (6.5%)	2 (8.0%)	8 (4.8%)	4 (9.1%)	0.704
HEPATIC ENCEPHALOPATHY	5 (3.3%)	0 (0.0%)	4 (2.4%)	1 (2.3%)	0.802
BEHAVIOURAL DISORDERS	2 (1.3%)	1 (4.0%)	1 (0.6%)	1 (2.3%)	0.489
CELIAC DISEASE	1 (0.7%)	0 (0.0%)	1 (0.6%)	1 (2.3%)	0.662
ARTHRITIS	9 (5.9%)	0 (0.0%)	10 (6.0%)	5 (11.4%)	0.291
METABOLIC SYNDROME	1 (0.7%)	0 (0.0%)	1 (0.6%)	1 (2.3%)	0.662
IDIOPATHIC CONSTIPATION	2 (1.3%)	2 (8.0%)	2 (1.2%)	2 (4.5%)	0.078
NON-ALCOHOLIC FATTY LIVER	4 (2.6%)	1 (4.0%)	7 (4.2%)	3 (6.8%)	0.631
NUMBER OF MICROBIOTA DYSBIOSIS RELATED DISEASE Median (Q1, Q3)	1.0 (1.0, 2.0)	2.0 (1.0, 2.0)	1.0 (1.0, 2.0)	1.0 (1.0, 2.0)	0.417
**ALCOHOL INTAKE > 50GR/DAY**	11 (4.8%)	1 (2.4%)	8 (3.0%)	3 (5.7%)	0.634
N-Miss	1	1	0	0	
**COLECTOMY OR ILEOSTOMY**	26 (11.4%)	6 (14.3%)	44 (16.7%)	5 (9.4%)	0.279
**CHOLECYSTECTOMY**	27 (11.8%)	5 (11.9%)	37 (14.0%)	7 (13.2%)	0.9
**IMMUNOCOMPROMISED**	87 (38.0%)	11 (26.2%)	131 (49.6%)	25 (47.2%)	0.007

CDI, *Clostridioides difficile* infection; NOCDI, Non *C. difficile diarrhoea*; RCDI, recurrent CDI.

N-Miss, Number of cases with no information.

Underlined values are significant p values (lower than 0.05).

The main risk factor for developing CDI was antibiotics in the month prior to sampling, with greater percentages in CDI and R-CDI: CDI, 91.5% (226/247); colonized, 86.0% (37/43); NOCDI, 83.5% (269/324); R-CDI, 93.4% (71/76) (p=0.007). Overall, the most used antibiotic groups in all patients were penicillins, third generation cephalosporins, quinolones and carbapenems (**Table 2**, **Supplementary Table 1**). With regard to the other CDI risk factors assessed, treatment with proton pump inhibitors and hospitalization were common in most patients. However, no significant differences were found between the groups for proton pump inhibitors, nasogastric tube use, mechanical ventilation, chemotherapy, radiotherapy, or dialysis (**Table 2**).

**Table 2:** CDI development risk factors.

	CDI N 255	COLONIZED N 44	NOCDI N 324	RCDI N 67	p.value
**ADMITTED PATIENT**	174 (68.2%)	24 (54.5%)	260 (80.2%)	25 (37.3%)	< 0.001
**ANTIBIOTIC TREATMENT**	233 (91.4%)	38 (86.4%)	269 (83.5%)	63 (94.0%)	0.012
N-Miss	0	0	2	0	
**NUMBER OF ANTIBIOTIC** Median (Q1, Q3)	2.0 (1.0, 4.0)	2.0 (1.0, 3.0)	3.0 (1.0, 4.0)	2.0 (1.0, 3.8)	0.251
**PROTON PUMP INHIBITOR TREATMENT**	220 (86.3%)	36 (83.7%)	269 (83.0%)	56 (84.8%)	0.76
N-Miss	0	1	0	1	
**NASOGASTRIC TUBE**	34 (13.3%)	7 (15.9%)	51 (15.7%)	6 (9.1%)	0.512
N-Miss	0	0	0	1	
**MECANIC VENTILATION**	37 (14.5%)	9 (20.5%)	53 (16.4%)	8 (12.1%)	0.622
N-Miss	0	0	0	1	
**SURGERY**	44 (17.3%)	11 (25.0%)	66 (20.4%)	12 (18.2%)	0.589
N-Miss	0	0	1	1	
**CHEMOTHERAPY OR RADIOTHERAPY**	42 (16.6%)	6 (13.6%)	78 (24.1%)	10 (14.9%)	0.06
N-Miss	2	0	0	0	
**DIALYSIS**	10 (3.9%)	4 (9.1%)	8 (2.5%)	5 (7.5%)	0.066
**IMMUNOSUPPRESS TREATMENT**	87 (34.3%)	15 (34.1%)	158 (48.8%)	22 (32.8%)	0.001
N-Miss	1	0	0	0	
**ANTIFUNGIC TREATMENT**	32 (12.6%)	5 (11.6%)	54 (16.7%)	8 (11.9%)	0.445
N-Miss	1	1	1	0	

CDI, *Clostridioides difficile* infection; NOCDI, Non *C. difficile diarrhoea*; RCDI, recurrent CDI. N-Miss, Number of cases with no information. CDI development risk factor in the month prior to sample collection.

Underlined values are significant p values (lower than 0.05).

As for severity, most episodes were mild (CDI, 63.6%; R-CDI, 65.8%). There were three cases of toxic megacolon and two cases of pseudomembranous colitis in the CDI group. Most CDI cases were hospital-onset, healthcare facility–associated (53.0% [131/247], while in the R-CDI patients, they were community-onset, healthcare facility–associated (50.0% [38/76]; p < 0.001). All groups contained patients who received treatment for CDI, as follows: CDI patients, 96.7% (238/246); colonized patients, 37.2% (16/43); NOCDI patients, 4.6% (15/324); and R-CDI patients 97.4% (74/76) (p<0.001). Most patients were treated with vancomycin. The recurrence rate was 25.7% in the primary CDI patients (53/206), and 30.0% in R-CDI (15/50). Five CDI patients (5/233; 2.1%) had a probable CDI-related death and four (4/233; 1.7%) had a clearly CDI-related death. In the R-CDI group, 3 (3/57; 5.3%) patients had a probable CDI-related death and 1 (1/57; 1.8%) a clearly CDI-related death (**Table 3**).

**Table 3:** Patients’evolution after sample collection.

	CDI N 255	COLONIZED N 44	NOCDI N 324	RCDI N 67	p.value
**CDI-RELATED DEATH**					<0.001
No_related	5 (2.0%)	3 (6.8%)	0	3 (4.5%)	
Probably_related	5 (2.0%)	0 (0.0%)	0	3 (4.5%)	
Clearly_related	4 (1.6%)	0 (0.0%)		1 (1.5%)	
**TREATMENT FAILURE**	5 (2.2%)	0 (0.0%)	0	0 (0.0%)	
N-Miss	26	32	324	10	
**30 DAYS MORTALITY**	19 (7.5%)	5 (11.4%)	11 (3.4%)	3 (4.5%)	0.053
**90 DAYS MORTALITY**	35 (13.7%)	7 (15.9%)	35 (10.8%)	7 (10.4%)	0.590
**RECURRENCE**	51 (25.8%)	0	0	20 (48.8%)	0.004
N-Miss	31	32	263	12	

CDI, *Clostridioides difficile* infection; NOCDI, Non *C. difficile diarrhoea*; RCDI, recurrent CDI.N-Miss, Number of cases with no information. CDI probably related death, Death was considered CDI-related when there were no other attributable causes and/or it occurred within 10 days after the diagnosis of CDI and/or was due to known complications of CDI.

Underlined values are significant p values (lower than 0.05).

### 3.2 Community structure (diversity)

**Figure 1:**
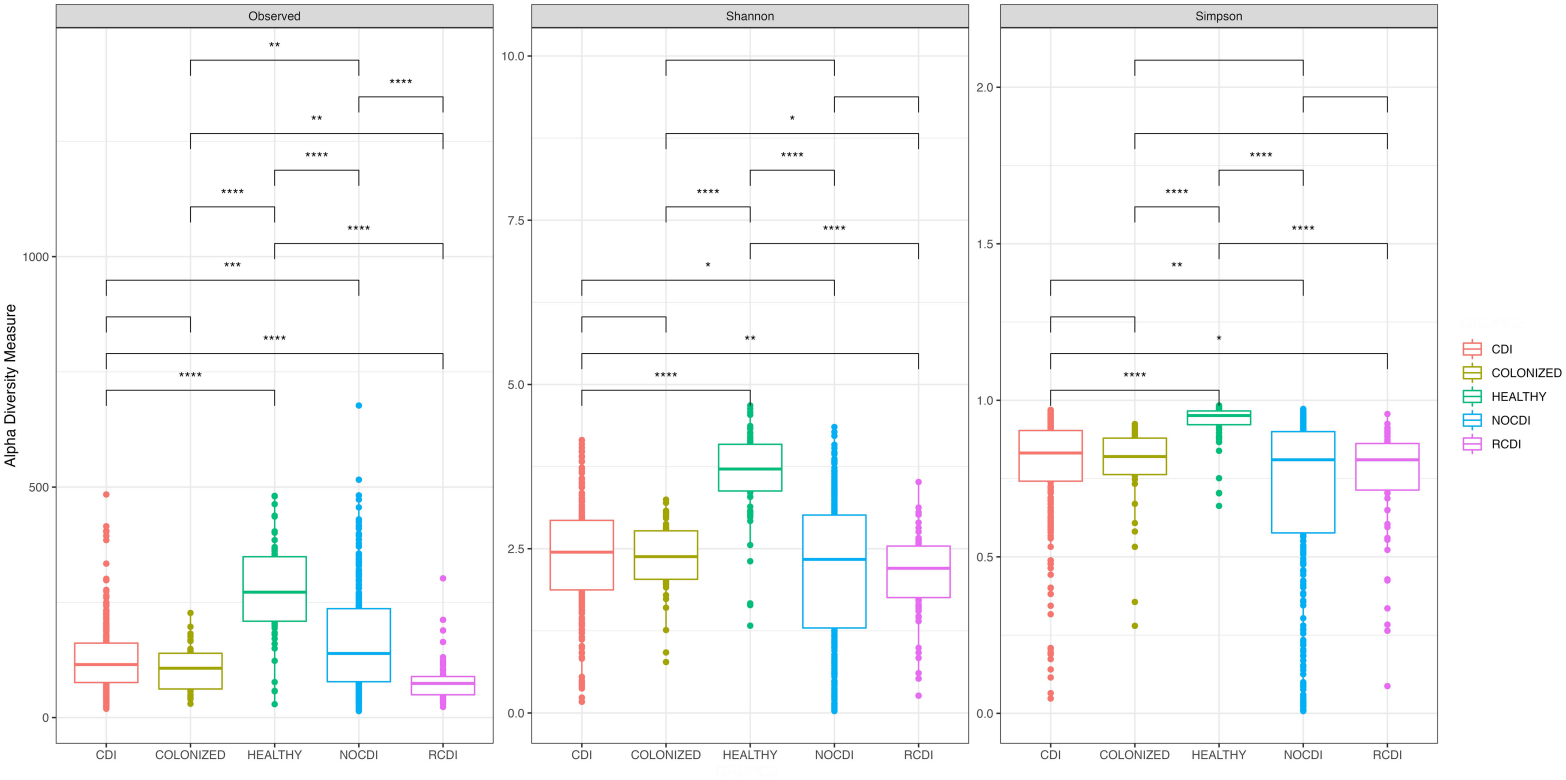
Box plot of Alpha diversity index (Shannon and Simpson) and Richness index (Observed) in all groups of patients. CDI, *Clostridioides difficile* infection; NOCDI, Non *C. difficile* diarrhoea; RCDI, recurrent CDI; Colonized, patients colonized with *C. difficile*; Healthy, Healthy subjects. *, p <= 0.05; **, p <= 0.01; ***, p <= 0.001; ****, p <= 0.0001.

Comparison of the 5 groups revealed significant differences in richness, alpha diversity, and evenness (all p < 0.001); these differences were maintained when the healthy individuals were removed from the comparison (all p < 0.05). In all cases, the R-CDI patients had the lowest richness, the lowest alpha diversity, and the lowest evenness (**Figure 1**).

When we stratified groups by age, all differences were maintained for those aged 16-69 years and for those over 69 years. When healthy individuals were removed from the comparison, there were significant differences in richness, alpha diversity, and evenness for those aged >69 years and in evenness for those aged 19-69 years. We found a series of significant differences, as follows: between CDI and NOCDI in richness, alpha diversity, and evenness (all p < 0.05); between CDI and R-CDI in richness and alpha diversity (all p < 0.05); between NOCDI and colonized patients in richness and evenness (p<0.05); and between R-CDI and colonized patients in richness and alpha diversity (p<0.05). No significant differences were found between CDI and colonized patients.

In all cases, we found significant differences between healthy individuals and each of the other groups, with higher richness, diversity, and evenness (p < 0.001) (**Figure 1**). Examination of each group individually revealed significant differences in alpha diversity and evenness among CDI with inflammatory bowel disease (IBD), with lower Shannon and Pielou indices. Differences in alpha diversity and evenness were also seen in patients who had taken a probiotic in the month prior to the episode, with lower Shannon, inverse Simpson, and Pielou indices than patients who had not taken probiotics (**Supplementary Table 2**). No significant differences in alpha diversity were seen in CDI, colonized patients, or R-CDI related to the presence or absence of toxin detected by Quick Check.

As for the NOCDI group, we found significant differences due to various factors, as follows: higher diversity and evenness values in patients over 69 years old; less diversity in patients who received antibiotics; higher diversity values in patients with underlying disease related to gut microbiota abnormalities and metabolic or cardiac disease; and lower diversity in patients with hematological disease, colectomy or ileostomy, colorectal cancer, or being immunosuppressed (**Supplementary Table 2**). Finally, in the R-CDI group, the group which presented the lowest microbiome diversity compared to the other CDI, colonized and healthy groups, we also found some differences within the R-CDI group. Patients presenting with colorectal cancer and colectomy or ileostomy had significantly higher diversity and evenness values than R-CDI patients without this condition, except for evenness in colorectal cancer (**Supplementary Table 2**).

**Figure 2:**
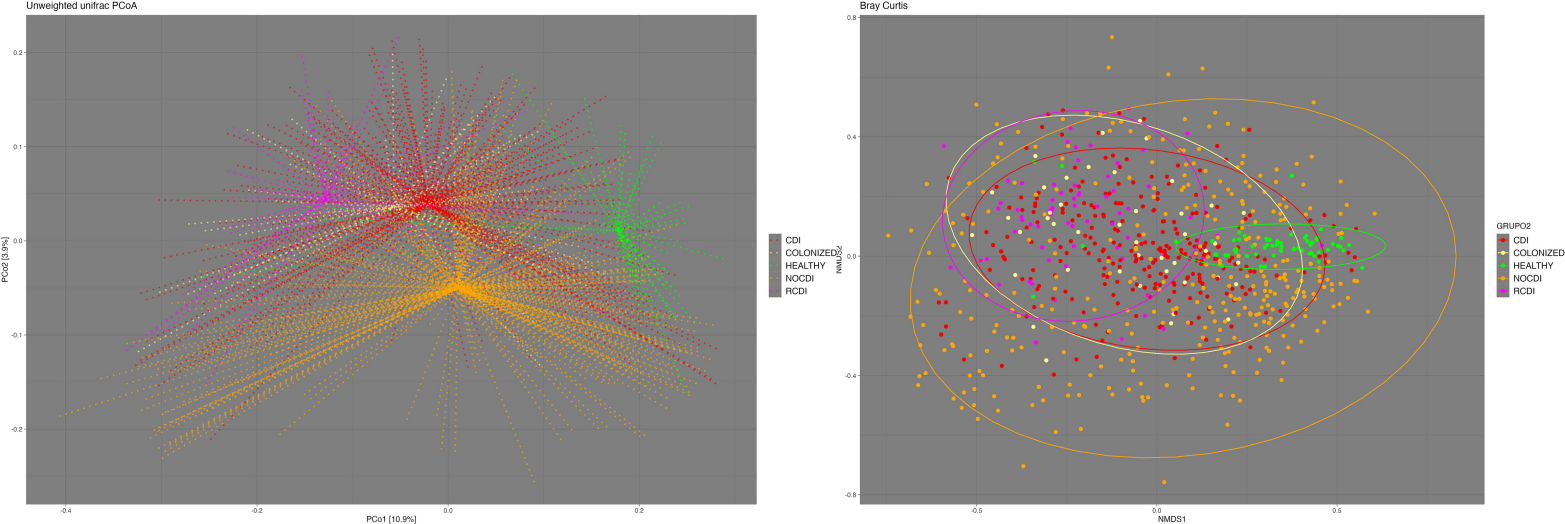
Beta- diversity. Left: Principal Coordinates Analysis (PCoA) based on unweighted UniFrac distances. Right: Non-metric multidimensional scaling (NMDS) plot based on the Bray-Curtis dissimilarity index. CDI, *Clostridioides difficile* infection; NOCDI, No *C. difficile* diarrhoea; RCDI, recurrent CDI; Colonized, patients colonized with *C. difficile*; Healthy, Healthy subjects.

We found significant differences in beta diversity between all the groups in general and in paired terms between all the groups except colonized patients and R-CDI (**Figure 2**). Regarding the homogeneity of the variance of the intra-patient samples, we found that the most homogenous patients were the healthy individuals, followed in decreasing order by CDI, R-CDI, colonized patients, and NOCDI patients, who were the most heterogeneous (**Figure 2**).

### 3.3 Community composition (relative abundance of taxa)

Regarding the relative abundance of the different taxonomic groups, we found that the major phylum, in all groups except CDI, was Firmicutes (healthy, 51.13%; NOCDI, 41.42%; colonized, 49.06%; and R-CDI, 39.86%), followed by Bacteroidetes (healthy, 31.23%; NOCDI, 36.37%; colonized, 25.09%; and R-CDI, 26.03%); in the CDI patients, the most common phylum was Bacteroidetes (39.59%), followed by Firmicutes (37.53%) (p<0.001). Differences in relative abundance between the groups were significant for Actinobacteria, Bacteroidetes, Firmicutes, Fusobacteria, and Proteobacteria. In the healthy individuals, we found a higher proportion of Actinobacteria and a lower proportion of Proteobacteria than in the other groups (p<0.001). When we compared the CDI, colonized, NOCDI, and R-CDI groups, we still found significant differences in Actinobacteria, Bacteroidetes, Firmicutes, and Proteobacteria (**Table 4**).

**Table 4:** Mean relative abundance (%) in each group at phylum level.

Phylum	CDI	COLONIZED	HEALTHY	NOCDI	RCDI	p.adj	p.adj without healthy
Actinobacteria	2.70	1.54	12.75	2.92	1.08	< 0.001	0.015
Bacteroidetes	**39.59**	25.09	31.23	36.37	26.03	< 0.001	0.003
Firmicutes	37.53	**49.06**	**51.13**	**41.42**	**39.86**	< 0.001	0.006
Fusobacteria	0.83	1.76	0.00	0.31	2.66	< 0.001	
Proteobacteria	16.20	17.11	2.96	16.90	24.77	< 0.001	0.001
Verrucomicrobia	3.15	5.43	1.93	2.09	5.59		

CDI, *Clostridioides difficile* infection; NOCDI, Non *C. difficile diarrhoea*; RCDI, recurrent CDI.

Bold values are the most abundant phyla in that group of patients.

**Figure 3:**
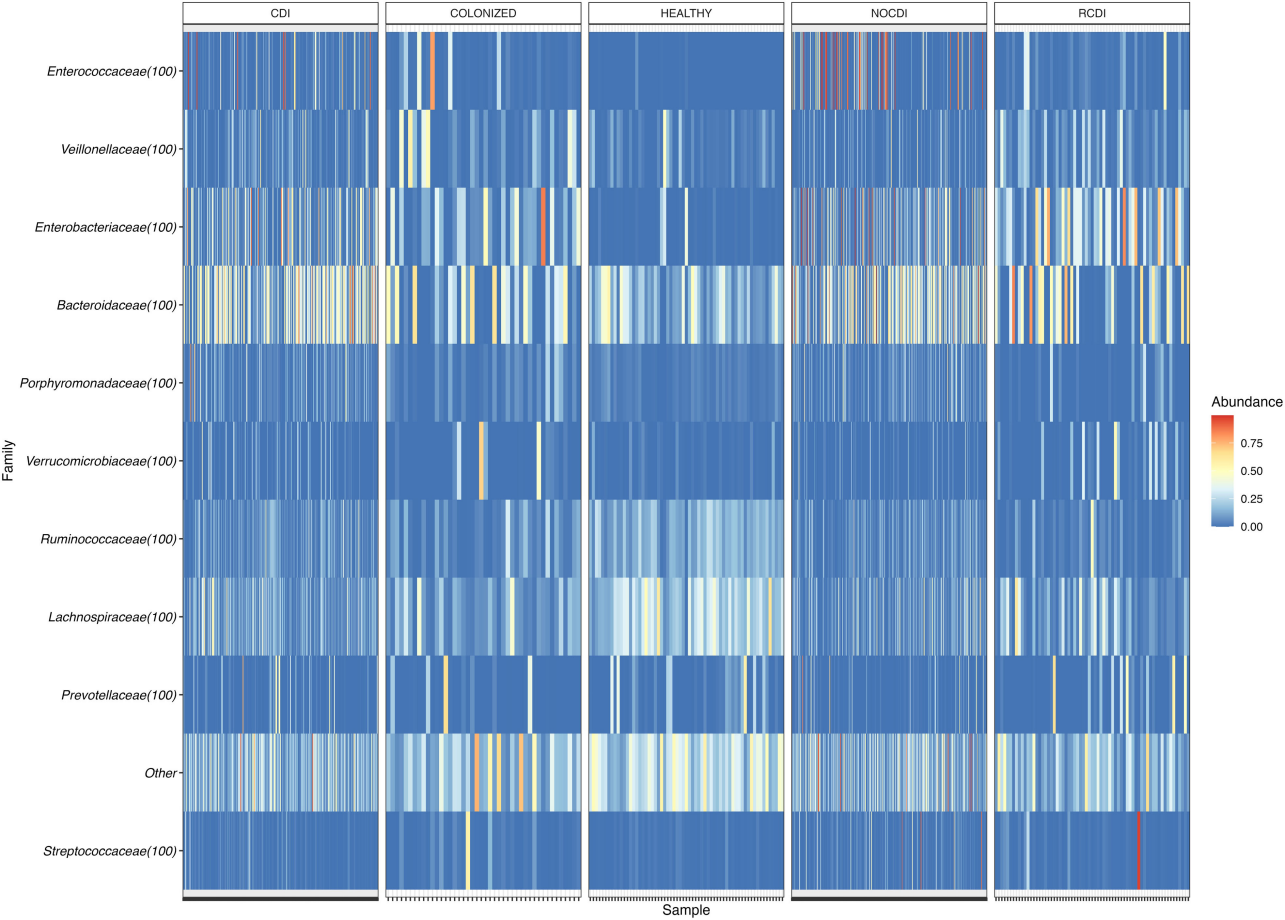
Heatmap of family relative abundance in each sample. CDI, *Clostridioides difficile* infection; NOCDI, Non *C. difficile* diarrhoea; RCDI, recurrent CDI; Colonized, patients colonized with *C. difficile*; Healthy, Healthy subjects.

Within the taxonomic family level, we found that Bacteroidaceae was the most abundant in the NOCDI, colonized, and CDI groups (NOCDI, 27.11%; colonized, 18.56%; and CDI, 29.99%), followed by Enterobacteriaceae (NOCDI, 15.29%; colonized, 15.26%; and CDI, 14.79%). However, in the healthy patients, the most abundant family was Lachnospiraceae (25.06%), followed by Bacteroidaceae (19.86%), and in the R-CDI patients, the most abundant was Enterobacteriaceae (23.06%), followed by Bacteroidaceae (20.14%). (**Table 5**) (**Figure 3**)

**Table 5:** Mean relative abundance (%) in each group at family level.

Phylum	Family	CDI	COLONIZED	HEALTHY	NOCDI	RCDI	p.adj	p.adj without healthy
Actinobacteria	Actinomycetaceae	0.10	0.05	0.04	0.34	0.03	0.010	< 0.001
	Bifidobacteriaceae	1.73	1.07	8.01	1.52	0.92	< 0.001	
	Coriobacteriaceae	0.85	0.41	4.70	1.02	0.11	< 0.001	< 0.001
Bacteroidetes	Bacteroidaceae	**29.99**	**18.56**	19.86	**27.11**	20.14	0.018	
	Porphyromonadaceae	4.86	3.46	2.96	4.72	2.76	< 0.001	< 0.001
	Rikenellaceae	2.63	2.05	2.93	3.12	0.99	< 0.001	< 0.001
Firmicutes	Bacillales_Incertae_Sedis_XI	0.02	0.01	0.01	0.02	0.01		< 0.001
	Enterococcaceae	7.25	6.97	0.26	14.75	4.20	< 0.001	
	Clostridiaceae_1	0.25	0.49	0.35	0.40	1.00	< 0.001	< 0.001
	Lachnospiraceae	10.31	10.97	**25.06**	8.07	11.96	< 0.001	< 0.001
	Peptostreptococcaceae	2.43	2.49	0.95	0.32	3.65	< 0.001	< 0.001
	Ruminococcaceae	5.70	6.98	14.99	5.24	3.17	< 0.001	0.009
	Erysipelotrichaceae	0.66	2.02	0.05	0.59	0.63	< 0.001	< 0.001
	Acidaminococcaceae	2.41	3.06	3.14	2.03	2.24	0.007	0.003
	Veillonellaceae	5.50	10.40	2.28	3.68	8.95	< 0.001	< 0.001
Fusobacteria	Fusobacteriaceae	0.83	1.76	0.00	0.31	2.66	0.003	
Proteobacteria	Sutterellaceae	0.37	0.22	0.47	0.38	0.45	< 0.001	
	Desulfovibrionaceae	0.51	0.68	0.27	0.45	0.44	0.034	
	Enterobacteriaceae	14.79	15.26	2.12	15.29	**23.06**	< 0.001	0.005

CDI, *Clostridioides difficile* infection; NOCDI, Non *C. difficile diarrhoea*; RCDI, recurrent CDI.

Bold values are the most abundant family in that group of patients.

Underlined values are p values lower than 0.001.

**Figure 4:**
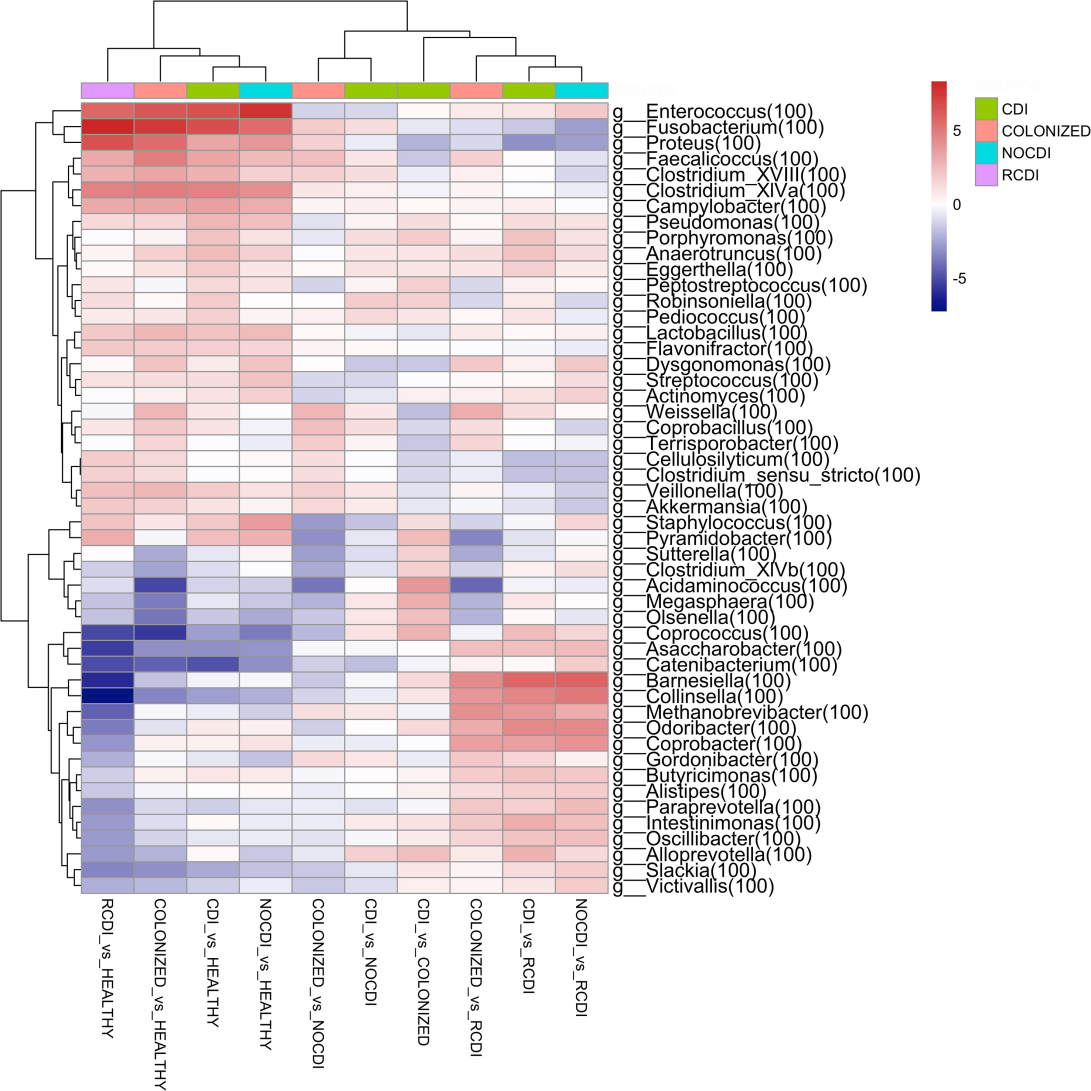
Genera heatmap of Log2 Fold Change represent differentially abundance genus between groups. CDI, *Clostridioides difficile* infection; NOCDI, Non *C. difficile* diarrhoea; RCDI, recurrent CDI; Colonized, patients colonized with *C. difficile*; Healthy, Healthy subjects.

When we reach the taxonomic level of genus, we find the following differences in abundance between the different groups (**Figure 4**).

#### 3.3.1 CDI vs healthy

For CDI patients vs. healthy, we found a significantly lower abundance in *Bifidobacterium, Collinsella, Slackia, Blautia, Butyrivibrio, Clostridium_XlVb, Coprococcus, Dorea, Fusicatenibacter, Ruminococcus, and Faecalibacterium, and a greater abundance of Staphylococcus, Enterococcus, Lactobacillus, Streptococcus, Parvimonas, Clostridium_XlVa, Robinsoniella, Peptostreptococcus, Clostridium_XVIII, Coprobacillus, Veillonella, Fusobacterium, Campylobacter, Proteus, Pseudomonas*, and *Akkermansia* (all p–0.05).

#### 3.3.2 CDI vs NOCDI

For CDI vs. NOCDI, we found genera with significant differences between the two groups. Decreases were recorded for *Dysgonomonas, Streptococcus, Clostridium_IV, Ruminococcus, Clostridium_XIVb, Megamonas, Acinetobacter*, and *Enterococcus*. Increases were recorded for *Blautia, Anaerostipes, Butyrivibrio, Clostridium_XlVa, Coprococcus, Robinsoniella, Butyricicoccus, Coprobacillus, Fusobacterium, Campylobacter*, and *Akkermansia* (all p<0.05).

#### 3.3.3 CDI vs colonized

For CDI vs. colonized, we recorded a reduction in *Faecalicoccus, Proteus, Weissella* and an increase in *Staphylococcus, Clostridium_XlVb, Coprococcus, Peptostreptococcus, Olsenella, Butyricicoccus, Robinsoniella, Megasphaera*, and *Alloprevotella* (all p<0.05).

#### 3.3.4 Colonized vs NOCDI

For colonized patients vs. NOCDI, we found significant differences, such as the decrease in the genera *Clostridium XIVb, Coprococcus, Staphylococcus*, and *Megasphaera* and a significant increase in *Coprobacillus, Faecalicoccus, Weisella*, and *Fusobacterium* (all p<0.05).

Of note, genera, such as *Enterococcus, Fusobacterium, Proteus, Faecalicoccus, Veillonella*, and *Akkermansia* and some *Clostridium* increased in all groups with respect to healthy individuals, and some of these genera maintained the same differences between the CDI and colonized groups vs. NOCDI. Other groups maintained the opposite pattern, and in the groups with *C. difficile*, several genera were less abundant, for example, *Bifidobacterium, Collinsella, Olsenella, Blautia, Butyrivibrio, Slackia, Coprococcus, Fusicatenibacter*, and *Megamonas.*

As for similarity between the OTUs of the different groups, we found that CDI and NOCDI shared the highest number of OTUs (4,523), followed by NOCDI and healthy individuals (3,296); the lowest values were found for R-CDI and healthy (1,172), colonized and healthy (1,128), and R-CDI and colonized (1,007).

### 3.4 Biomarker analysis: linear discrimination analysis and random forest analysis

The biomarker search was based on a linear discrimination analysis, which revealed various genera to be possible discriminating biomarkers for patients with high scores (> 3).

**Figure 5:**
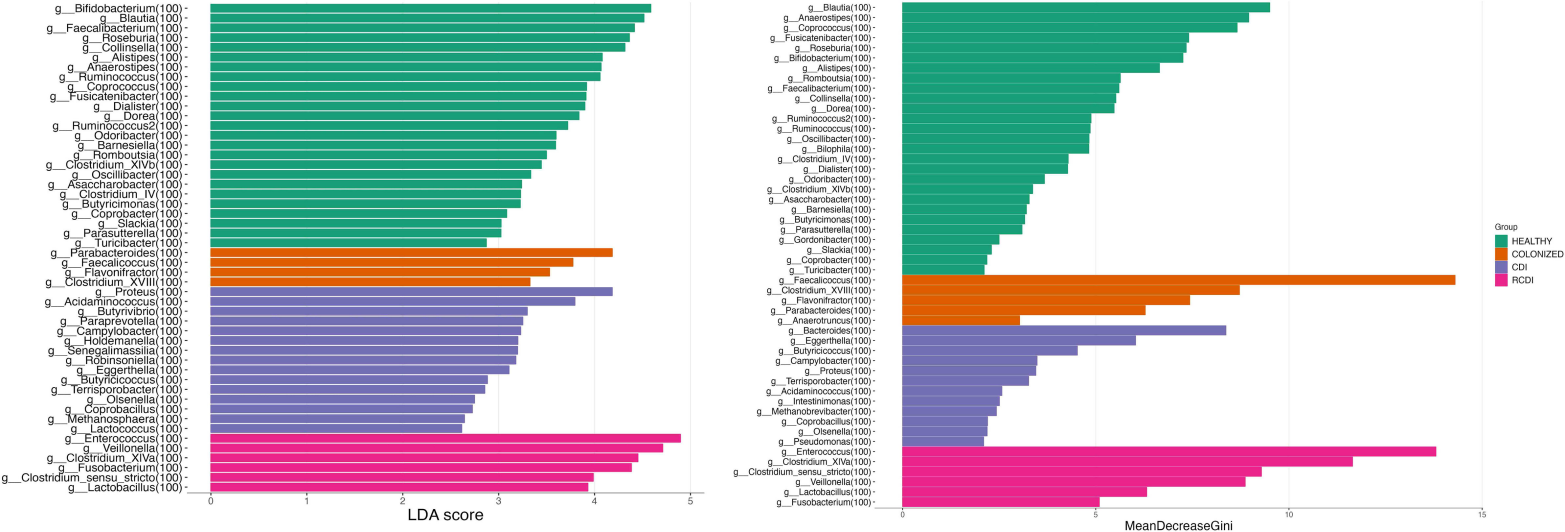
List of possible genera biomarkers that enable discrimination between groups. Left (Linear discriminant analysis (LDA). Right Random Forest (RF)). CDI, *Clostridioides difficile* infection; NOCDI, Non *C. difficile* diarrhoea; RCDI, recurrent CDI; Colonized, patients colonized with *C. difficile*; Healthy, Healthy subjects.

**Figure 6:**
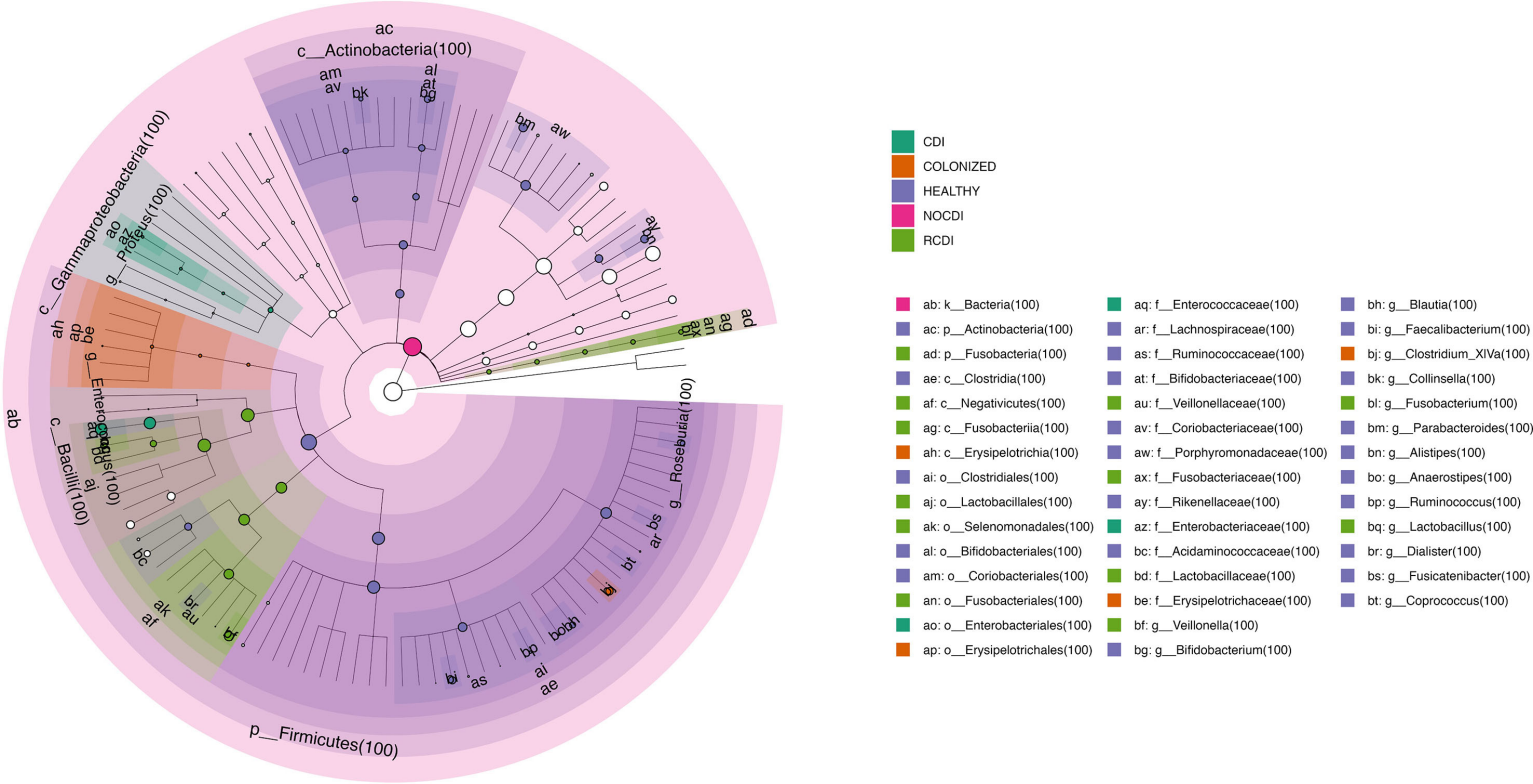
Cladogram with taxa different abundance between groups CDI, *Clostridioides difficile* infection; NOCDI, Non *C. difficile* diarrhoea; RCDI, recurrent CDI; Colonized, patients colonized with *C. difficile*; Healthy, Healthy subjects.

The potential biomarkers identified for colonization were *Parabacteroides, Faecalicoccus, Flavonifractor*, and *Clostridium_XVIII*. For CDI group, the biomarkers were *Bacteroides, Proteus, Paraprevot ella, Butyrivibrio, Senegalimassilia, Holdemanela, Robinsoniella*, and *Eggerthellas*. In R-CDI patients, they were *Veillonella, Fusobacterium, Lactobacillus, Enterococcus, Clostridium_XIVa*, and *Clostridium_sensu_stricto*. Finally, in the healthy patients, we identified, among others, *Bifidobacterium, Blautia, Faecalibacterium*, and *Roseburia* (**Figures 5**, **6**).

We also performed a random forest analysis, which identified the same discriminating genera for each patient (**Figure 5**).

## 4 Discussion

In the present study, we outlined the differences in the microbiota of five groups of patients (healthy, colonized, CDI, NOCDI, and R-CDI) from a large cohort, finding marked differences that could be useful as diagnostic markers of true CDI infection.

Several studies have characterized microbiota in patients with CDI, although the groups compared were very heterogeneous. Some studies based their control group on “healthy” individuals, which is a confusing term, since “healthy” can include patients with other diseases but not CDI or diarrhea, patients receiving different treatments for their underlying disease, persons without diarrhea who have not taken antibiotics, and even stool donors for fecal transplantation (Schubert et al., 2014; Milani et al., 2016; Amrane et al., 2019; Sánchez-Pellicer et al., 2021). We compared various groups, including both healthy individuals and patients with diarrhea in whom *C. difficile* has been ruled out but who share diseases, treatments, and other characteristics that may more closely resemble those of CDI patients.

The comparison with healthy individuals revealed a considerable difference in the state of the intestinal microbiota compared with patients with gastrointestinal disorders, whether due to *C. difficile* or not, namely, greater richness, alpha diversity, and evenness. These differences are also evident in beta diversity, where the healthy individuals group differs significantly from all the others.

It should also be noted that we did find differences in alpha diversity and richness between the CDI and NOCDI groups, in contrast with observations reported elsewhere, perhaps because our sample was larger than those of other studies (Sangster et al., 2016; Jeon et al., 2019; Herrera et al., 2021). Most studies comparing the microbiota of patients with CDI with that of patients with diarrhea due to other causes and with the microbiota of persons colonized by *C. difficile* include a low number of individuals for each study group (Zhang et al., 2015; Allegretti et al., 2016; Milani et al., 2016; Sangster et al., 2016; Jeon et al., 2019; Herrera et al., 2021). This observation is very important, since the intestinal microbiome is constantly changing due to factors such as diet, environmental factors, sports, and medication (Dominianni et al., 2015; Rashid et al., 2015; Mohr et al., 2020). Therefore, in order to obtain more reliable results, it is necessary to include a considerable number of persons in each group, as in our study.

Consistent with the literature, the lowest diversity and richness values were recorded for patients with R-CDI (Chang et al., 2008; Allegretti et al., 2016; Gazzola et al., 2020). Both our data and that of other authors show the existing damage to the microbiota or dysbiosis in patients with diarrhea due to *C. difficile* or other causes and that of patients colonized by *C. difficile* (Sangster et al., 2016; Sokol et al., 2018; Crobach et al., 2020; Sánchez-Pellicer et al., 2021; Wan et al., 2022). In addition, the greater damage to the microbiota in patients with repeated episodes of CDI is highlighted. In this study, we observed that patients with CDI and IBD presented less alpha diversity and evenness than CDI patients without IBD.

Interestingly, we found that among patients with diarrhea, there were markedly significant differences in beta diversity between those with diarrhea due to CDI and those with diarrhea due to other causes (NOCDI). Some authors have reported similar findings (Schubert et al., 2014; Sangster et al., 2016; Han et al., 2020), whereas others have not (Antharam et al., 2013; Jeon et al., 2019; Gazzola et al., 2020; Herrera et al., 2021). Moreover, we observed that beta diversity differed significantly in patients with CDI and patients colonized by *C. difficile*. This finding has not been reported in the few studies carried out that include these groups, probably owing to the limited number of samples analyzed (Han et al., 2019; Crobach et al., 2020; Sánchez-Pellicer et al., 2021).

In agreement with the literature (Crobach et al., 2020), we observed that alpha and beta diversity were significantly altered by specific CDI risk factors (antibiotic treatment in the previous month, previous episodes of CDI, treatment with proton pump inhibitors or probiotics in the previous month, nasogastric tube in the previous month), colorectal cancer, solid organ transplant, neoplasia, and hematological disease.

As for differences in abundance, we found that, except in patients with CDI, the most abundant phylum in all five groups was Firmicutes followed by Bacteroidetes. In the CDI group, this order was inverted, with Bacteroidetes being the most abundant phylum, as reported elsewhere (Antharam et al., 2013; Sánchez-Pellicer et al., 2021), although the opposite has also been observed (Zhang et al., 2015; Han et al., 2019; Han et al., 2020). These contradictions in the literature may be due to the different methodologies used for sample extraction, amplification, and analysis, together with the limited number of samples processed. Consistent with other observations in the literature, we also found a decrease in Actinobacteria and an increase in Proteobacteria in patients with CDI compared to healthy individuals (Amrane et al., 2019; Han et al., 2019; Han et al., 2020; Sánchez-Pellicer et al., 2021). When we assessed the genera by groups, we found that *Ruminococcus*, *Clostridium*_IV, *Streptococcus*, *Acinetobacter*, and *Megamonas* decreased in the CDI group compared to CDI. The opposite occurred *Blautia* and *Coprococcus*, which increased compared to NOCDI. Other genera that were more abundant in CDI compared to both healthy individuals and NOCDI were *Lactobacillus, Clostridium_XIVa, Robinsoniella, Coprobacillus, Fusobacterium, Campylobacter*, and *Akkermansia*. The decrease in butyrate-producing bacteria, such as *Ruminococcus*, and the increase in genera of lactic acid–producing bacteria, such as *Blautia*, *Lactobacillus*, and *Fusobacterium*, has been associated with an increased risk of CDI (Vakili et al., 2020).

Significant differences were found for the presence of genera observed in CDI patients versus NOCDI or those for CDI patients versus colonized patients. This enabled us to carry out an exhaustive search for microbial biomarkers that could help respond to the as yet unresolved need to identify diarrhea truly caused by *C. difficile*. Few studies have attempted to outline these differences.

Among the few studies comparing the intestinal microbiota of patients with CDI and *C. difficile* colonization, the number of patients included is lower than in ours, with very few significant differences between the two groups (Zhang et al., 2015; Han et al., 2019; Crobach et al., 2020; Sánchez-Pellicer et al., 2021). Furthermore, the study conducted by Han et al. did not discriminate between toxigenic and non-toxigenic *C. difficile* strains, meaning that the *C. difficile* study groups included patients with toxigenic and non-toxigenic *C. difficile*, thus making it difficult to interpret and extrapolate their results (Han et al., 2019). We identified a series of changes in the microbiota that enabled us to outline the differences between colonized patients and those with true CDI infection. In CDI patients, we observed two genera that decreased significantly, namely, *Faecalicoccus* and *Proteus*, both of which are increased in colonized patients. We also found a significant increase in *Clostridium_XIVb, Coprococcus, Staphylococcus*, and *Robinsoniella* among patients with true CDI. These differences could facilitate appropriate categorization of these groups of patients, which, as mentioned above, remains unresolved.

To the best of our knowledge, this is the first study to have carried out such a broad comparison to identify biomarkers that differentiate between so many subgroups of patients, especially between CDI patients and colonized patients. The few previous studies on this area focused on finding biomarkers to be able to distinguish between CDI and healthy individuals (Antharam et al., 2013), CDI and NOCDI (Schubert et al., 2014; Herrera et al., 2021), and CDI and R-CDI (Allegretti et al., 2016).

Our results were also consistent with those of previous studies. We observed a decrease in certain families or genera involved in the production of butyric acid, such as Ruminococcaceae (*Faecalibacterium* and *Ruminococcus*) and Lachnospiraceae (*Blautia* and *Coprococcus*), both in CDI patients and in colonized patients (Antharam et al., 2013; Zhang et al., 2015; Milani et al., 2016). For CDI and colonized patients, we also observed a decrease in *Bifidobacterium*, a genus with anti-inflammatory and antimicrobial properties. (Imaoka et al., 2008) We identified a series of genera that could be of use as biomarkers. These included *Parabacteroides*, *Faecalicoccus*, *Flavonifractor*, and *Clostridium_XVIII* for the colonized group, and *Veillonella*, *Fusobacterium*, *Lactobacillus, Enterococcus, Clostridium_XIVa*, and *Clostridium_sensu_sricto* for the R-CDI group. We also identified genera that better define true CDI infection, such as *Bacteroides*, *Proteus*, *Paraprevotella*, *Robinsoniella*, and *Eggerthella*.

Our study is limited by its single-center design, which requires our results to be validated in multicenter studies. However, as our sample is the largest microbiome analysis of CDI to date, our results can be considered robust. Basing our study on 5 different groups of patients enabled us to analyze the different microbiota profiles and thus better characterize those microbiota abnormalities caused solely by *C. difficile* infection. We also separated primary CDI from R-CDI, where the progressive damage that *C. difficile* produces in the intestinal microbiota is evident.

In conclusion, we found several genera that could be used as biomarkers to differentiate between *C. difficile*–colonized patients, true CDI episodes, and diarrhea due to other causes. Our approach will enable us to improve the diagnosis, management, and treatment of CDI. Further research is warranted.

## Data availability statement

The datasets presented in this study can be found in online repositories. The names of the repository/repositories and accession number(s) can be found below: https://www.ebi.ac.uk/ena, PRJEB57947.

## Ethics statement

The studies involving human participants were reviewed and approved by Ethics Committee of Hospital General Universitario Gregorio Marañón in Madrid (number MICRO.HGUGM.2016-029). Written informed consent to participate in this study was provided by the participants’ legal guardian/next of kin.

## Author contributions

ER and EB designed the study. SV-C performed the collection of data and samples. SV-C and NG performed the data analysis. SV-C, ER, EB, MO, LA, MM, PM, contributed to data interpretation. SV-C wrote the first draft of the manuscript. SV-C, ER, EB, MO, LA, MM, PM, AF and LV, contributed with the intellectual content and approved the final draft. SV-C, ER, and EB had access to and verified the underlying study data. All authors had full access to all the data in the study and had final responsibility for the decision to submit for publication.

## Group members of HGUGM Microbiome Group

Ana I. Fernández, Carmen Moreno, Celso Arando, Darío García de Viedma, Elena Reigadas, Francisco Fernández-Avilés, Javier Bermejo, Javier Vaquero, Lorena Cussó, Manuel Desco, Mara Perellada, Marta Fernández, Nuria López, Rafael Correa, Silvia Vázquez-Cuesta.
