# Supplementary material for: Characterization of the gut microbiome of patients with Clostridioides difficile infection, patients with non–C. difficile diarrhea, and C. difficile–colonized patients
Source: Front Cell Infect Microbiol. 2023 Apr 12;13:1130701. doi: 10.3389/fcimb.2023.1130701 (PMC10130453; doi:10.3389/fcimb.2023.1130701)
Supplement: Supplementary file 1 [file DataSheet_1.pdf]

Supplementary Material

Characterization of the Gut Microbiome of Patients with *Clostridioides difficile* Infection, Patients with non–*C. difficile* Diarrhea, and *C. difficile*–Colonized Patients

Silvia Vázquez-Cuesta*, Laura Villar, Nuria Lozano García, Ana I Fernández, María Olmedo, Luis Alcalá, Mercedes Marín, Patricia Muñoz, Emilio Bouza, Elena Reigadas

*** Correspondence:** Silvia Vázquez-Cuesta: silviavazquez.lab@gmail.com

1 Supplementary Tables

Supplementary Table 1. Antibiotic treatment received one month prior to sample collection.

	CDI N 255	COLONIZED N 44	NOCDI N 324	RCDI N 67	p.value
**TOTAL DDDs** Median (Q1, Q3)	12.2 (6.4, 22.2)	15.2 (6.0, 22.9)	10.6 (4.7, 22.5)	10.0 (4.5, 15.6)	0.237
N-Miss	27	2	57	5	
**DDDs ANAEROBIC** Median (Q1, Q3)	7.0 (3.0, 12.4)	7.9 (5.0, 13.0)	6.0 (2.6, 10.9)	6.0 (2.0, 10.8)	0.533
N-Miss	99	14	126	36	
**SULPHONAMIDE**	34 (14.8%)	7 (16.3%)	66 (24.5%)	8 (12.9%)	0.022
N-Miss	26	1	55	5	
**QUINOLONES**	74 (32.3%)	15 (34.9%)	87 (32.3%)	21 (33.9%)	0.983
N-Miss	26	1	55	5	
**RIFAMYCIN**	12 (5.2%)	3 (7.0%)	13 (4.8%)	1 (1.6%)	0.59
N-Miss	26	1	55	5	
**CARBAPENEMS**	70 (30.6%)	10 (23.3%)	68 (25.3%)	15 (24.2%)	0.487
N-Miss	26	1	55	5	
**GLUCOPEPTIDES**	33 (14.4%)	10 (23.3%)	54 (20.1%)	34 (54.8%)	< 0.001
N-Miss	26	1	55	5	
**1ST GENERATION CEPHALOSPORINS**	21 (9.2%)	4 (9.3%)	22 (8.2%)	1 (1.6%)	0.263
N-Miss	26	1	55	5	
**2ND GENERATION CEPHALOSPORINS**	11 (4.8%)	0 (0.0%)	3 (1.1%)	3 (4.8%)	0.04
N-Miss	26	1	55	5	
**3RD GENERATION CEPHALOSPORINS**	87 (38.0%)	13 (30.2%)	85 (31.6%)	11 (17.7%)	0.023
N-Miss	26	1	55	5	
**4TH GENERATION CEPHALOSPORINS**	11 (4.8%)	4 (9.3%)	11 (4.1%)	1 (1.6%)	0.298
N-Miss	26	1	55	5	
**5TH GENERATION CEPHALOSPORINS**	4 (1.7%)	0 (0.0%)	2 (0.7%)	0 (0.0%)	0.468

N-Miss	26	1	55	5	
**PENICILLINS**	95 (41.5%)	20 (46.5%)	138 (51.3%)	19 (30.6%)	0.014
N-Miss	26	1	55	5	
**LIPOPEPTIDES**	4 (1.7%)	0 (0.0%)	6 (2.2%)	1 (1.6%)	0.784
N-Miss	26	1	55	5	
**AMINOGLYCOSIDES**	7 (3.1%)	4 (9.3%)	18 (6.7%)	2 (3.2%)	0.147
N-Miss	26	1	55	5	
**PHOSPHONATES**	8 (3.5%)	5 (11.6%)	9 (3.3%)	4 (6.5%)	0.064
N-Miss	26	1	55	5	
**NITROIMIDAZOLE**	24 (10.5%)	8 (18.6%)	47 (17.5%)	13 (21.0%)	0.072
N-Miss	26	1	55	5	
**MACROLIDES**	10 (4.4%)	4 (9.3%)	35 (13.0%)	3 (4.8%)	0.005
N-Miss	26	1	55	5	
**OXAZOLIDINONES**	18 (7.9%)	3 (7.0%)	29 (10.8%)	3 (4.8%)	0.398
N-Miss	26	1	55	5	
**LINCOSAMIDES**	5 (2.2%)	2 (4.7%)	13 (4.8%)	0 (0.0%)	0.157
N-Miss	26	1	55	5	
**MONOBACTAMS**	2 (0.9%)	2 (4.7%)	4 (1.5%)	1 (1.6%)	0.318
N-Miss	26	1	55	5	
**TETRACYCLINES**	2 (0.9%)	0 (0.0%)	1 (0.4%)	0 (0.0%)	0.738
N-Miss	26	1	55	5	
**CEPHALOSPORINS**	122 (53.3%)	19 (44.2%)	115 (42.9%)	16 (25.8%)	0.001
N-Miss	26	1	56	5	
**BETALACTAMICS**	196 (85.6%)	35 (81.4%)	221 (82.5%)	33 (53.2%)	< 0.001
N-Miss	26	1	56	5	
**ANAEROBICS**	157 (68.6%)	31 (72.1%)	200 (74.6%)	32 (51.6%)	0.005
N-Miss	26	1	56	5	

CDI: *Clostridioides difficile* infection; NOCDI: No *C. difficile* diarrhoea; RCDI: recurrent CDI. . Q1: ;Q3: Quartile 1, Quartile 3; N-Miss: Number of cases with no information. Defined Daily Dose (DDD) is defined by "WHO Collaborating Centre for Drug Statistics Methodology" as the assumed average maintenance dose per day for a drug used for its main indication in adults. Anaerobics including penicillins, with beta-lactamase inhibitors too, lincosamides and metronidazole

Supplementary Table 2. Intra-group alpha diversity and richness index

	Shannon	p. value	Invsimpson	p. value	Pielou	p. value	sobs	p. value
**CDI**								
**IBD**		0.06		0.21		0.024		0.088
**Yes**	2.004 (0.907)		6.080 (5.704)		0.415 (0.152)		96.826 (59.905)	
**No**	2.441 (0.814)		8.226 (6.110)		0.504 (0.139)		139.618 (91.103)	
**Probiotic treatment**		0.01		0.066		0.012		0.108
**Yes**	1.897 (0.965)		5.241 (5.050)		0.410 (0.177)		100.780 (69.834)	
**No**	2.435 (0.794)		8.063 (5.972)		0.502 (0.138)		134.450 (81.399)	
**Toxin B PCR Cycles**		0.344		0.27		0.03		0.024
**<23**	2.330 (0.577)		6.984 (3.679)		0.508 (0.138)		135.317 (82.518)	
**23-30**	2.463 (0.832)		8.412 (6.459)		0.443 (0.183)		153.743 (96.776)	
**>30**	2.281 (1.022)		7.419 (6.605)		0.497 (0.144)		132.548 (81.279)	
**COLONIZED**								
**IBD**		0.005		0.105		0.002		0.26
**Yes**	0.777 (NA)		1.391 (NA)		0.203 (NA)		47.661 (NA)	
**No**	2.403 (0.515)		6.540 (2.991)		0.516 (0.090)		111.208 (53.924)	
**Diabetes Mellitus**		0.036		0.028		0.031		0.024
**Yes**	2.575 (0.556)		7.616 (3.339)		0.548 (0.085)		131.600 (59.667)	
**No**	2.080 (0.556)		4.944 (2.191)		0.456 (0.113)		83.822 (35.405)	
**NOCDI**								
**AGE GROUP**		0.003		0.011		0.021		<0.001
**<19**	1.771 (0.590)		3.720 (1.558)		0.409 (0.130)		92.624 (50.745)	
**19-69**	1.975 (1.126)		6.255 (5.380)		0.398 (0.216)		150.748 (108.925)	
**>69**	2.405 (1.003)		8.138 (6.698)		0.466 (0.176)		201.163 (117.663)	
**ANTIBIOTIC TREATMENT**		0.018		0.014		0.012		0.12
**Yes**	2.125 (1.083)		6.751 (5.753)		0.421 (0.200)		170.158 (114.741)	
**No**	2.559 (0.933)		9.329 (7.507)		0.505 (0.158)		200.996 (117.422)	
**MICROBIOTA-RELATED DISEASE**		<0.001		0.002		0.003		0.005
**Yes**	2.350 (1.054)		8.015 (6.535)		0.460 (0.191)		189.221 (120.031)	
**No**	1.899 (1.048)		5.565 (4.915)		0.385 (0.199)		148.343 (102.670)	
**HEMATOLOGICAL MALIGNANCIES**		<0.001		0.003		0.007		<0.001
**Yes**	1.705 (1.065)		4.998 (4.567)		0.372 (0.215)		114.868 (81.874)	
**No**	2.322 (1.037)		7.723 (6.351)		0.450 (0.188)		191.105 (118.206)	
**METABOLIC DISEASE**		0.008		0.004		0.014		0.01
**Yes**	2.401 (1.054)		8.443 (6.646)		0.469 (0.192)		196.883 (116.351)	
**No**	2.045 (1.064)		6.254 (5.568)		0.409 (0.196)		159.480 (112.878)	
**CARDIOLOGICAL DISEASE**		<0.001		0.002		<0.001		<0.001
**Yes**	2.347 (1.041)		7.968 (6.573)		0.463 (0.184)		191.346 (117.648)	
**No**	1.880 (1.068)		5.520 (4.721)		0.376 (0.208)		142.056 (104.563)	

**COLECTOMY OR ILEOSTOMY**		<0.001		0.002		0.007		<0.001
**Yes**	1.687 (0.953)		4.480 (3.422)		0.360 (0.193)		118.718 (73.995)	
**No**	2.286 (1.068)		7.653 (6.383)		0.447 (0.194)		185.483 (119.180)	
**COLORRECTAL CARCINOMA**		0.019		0.039		0.026		0.043
**Yes**	1.762 (1.034)		4.815 (4.091)		0.359 (0.182)		131.576 (88.471)	
**No**	2.412 (1.040)		8.352 (6.661)		0.470 (0.189)		195.288 (121.521)	
**IMMUNOSUPPRESSED PATIENT**		<0.001		<0.001		<0.001		<0.001
**Yes**	1.924 (1.054)		5.665 (4.643)		0.392 (0.200)		139.949 (97.350)	
**No**	2.445 (1.029)		8.562 (6.984)		0.473 (0.185)		208.245 (122.155)	
**RCDI**								
**COLECTOMY OR ILEOSTOMY**		0.011		< 0.001		0.142		< 0.001
**Yes**	2.798 (0.505)		10.895 (7.503)		0.567 (0.101)		133.537 (52.232)	
**No**	2.084 (0.582)		5.543 (2.546)		0.479 (0.127)		77.167 (32.290)	
**COLORRECTAL CARCINOMA**		0.016		0.002		0.156		0.002
**Yes**	2.798 (0.505)		10.895 (7.503)		0.567 (0.101)		77.640 (34.032)	
**No**	2.070 (0.622)		5.598 (2.684)		0.474 (0.138)		133.537 (52.232)	

This table shows the subgroups in which some significant differences were found.CDI: *Clostridioides difficile* infection; NOCDI: No *C. difficile* diarrhoea; RCDI: recurrent CDI; IBD: Inflammatory bowel disease; Invsimpson: Inverse Simpson index; sobs: Observed species index; Shannon: Shannon diversity index; Pielou: Pielou's evenness index.
